# Increased permeability-oedema and atelectasis in pulmonary dysfunction after trauma and surgery: a prospective cohort study

**DOI:** 10.1186/1471-2253-7-7

**Published:** 2007-07-09

**Authors:** AB Johan Groeneveld

**Affiliations:** 1 Department of Intensive Care, Institute for Cardiovascular Research, Vrije Universiteit Medical Center, Amsterdam, The Netherlands

## Abstract

**Background:**

Trauma and surgery may be complicated by pulmonary dysfunction, acute lung injury (ALI) and acute respiratory distress syndrome (ARDS), but the mechanisms are incompletely understood.

**Methods:**

We evaluated lung capillary protein permeability non-invasively with help of the ^67^Ga-transferrin pulmonary leak index (PLI) technique and extravascular lung water (EVLW) by the transpulmonary thermal-dye dilution technique in consecutive, mechanically ventilated patients in the intensive care unit within 24 h of direct, blunt thoracic trauma (n = 5, 2 with ARDS), and within 12 h of indirect trauma by transhiatal oesophagectomy (n = 8), abdominal surgery for cancer (n = 6) and bone surgery (n = 4). We studied transfusion history, haemodynamics, oxygenation and mechanics of the lungs. The lung injury score (LIS, 0–4) was calculated. Plain radiography was also done to judge densities and atelectasis.

**Results:**

The PLI and EVLW were elevated above normal in 61 and 30% of patients, respectively, and the PLI directly related to the number of red cell concentrates given (r_s _= 0.69, P < 0.001), without group differences. Oxygenation, lung mechanics, radiographic densities and thus the LIS (1.0 [0.25–3.5]) did not relate to PLI and EVLW. However, groups differed in oxygenation and airway pressures and impaired oxygenation related to the number of radiographic quadrants with densities (r_s _= 0.55, P = 0.007). Thoracic trauma patients had a worse oxygenation requiring higher airway pressures and thus higher LIS than the other patient groups, unrelated to PLI and EVLW but attributable to a higher cardiac output and thereby venous admixture. Finally, patients with radiographic signs of atelectasis had more impaired oxygenation and more densities than those without.

**Conclusion:**

The oxygenation defect and radiographic densities in mechanically ventilated patients with pulmonary dysfunction and ALI/ARDS after trauma and surgery are likely caused by atelectasis rather than by increased permeability-oedema related to red cell transfusion.

## Background

Trauma and major surgery may be complicated by pulmonary dysfunction and acute lung injury (ALI)/acute respiratory distress syndrome (ARDS) [1]. After vascular or cardiac surgery, for instance, ischemia/reperfusion and pro-inflammatory responses may result in increased permeability followed by oedema in the lungs [2,3]. In these studies, we used the non-invasive ^67^Ga-transferrin pulmonary leak index (PLI) measured at the bedside to assess permeability, and the transpulmonary thermal-dye dilution for assessment of accessible extravascular lung water (ELVW) and thereby to indirectly estimate atelectasis, which can be hard to differentiate from oedema, even sometimes by computer tomography (CT) scanning [2-10]. Indeed, ALI/ARDS, a continuum from moderate to severe pulmonary injury with increased permeability-oedema [1,5,11,12] can be accompanied by atelectasis, contributing to ventilatory and radiographic abnormalities. It may be important to differentiate atelectasis from increased-permeability oedema because of differing therapeutic consequences [1,6,9,13-16].

Mechanisms of postoperative pulmonary dysfunction and ALI/ARDS may depend on the type of trauma and surgery. Thoracic trauma and pulmonary contusion may result in direct ALI/ARDS [12,17,18], and some authors suggested a major contribution by atelectasis [15,19]. Transhiatal surgery for cancer of the oesophagus carries a >20% risk of postoperative respiratory complications and mechanisms may be multiple [20-22]. Other types of abdominal surgery, including liver resections, may result in postoperative pulmonary complications in 10 to 70% of patients, with atelectasis, pneumonia or oedema/ALI [9,13,16,23,24]. Pulmonary complications are frequent after bone and spinal surgery and, again, causes may be multiple [25,26,34]. In contrast, transfusion of blood products during and after trauma and surgery may be a common risk factor for ALI/ARDS and contribute to transfusion-related lung injury (TRALI), a form of indirect ALI/ARDS [25,27,28]. On the other hand, anaesthesia and subsequent surgery may result in atelectasis and thereby in hypoxemia, and may be another potentially common factor among trauma and surgery types [9,29]. Taken together, the pathogenesis and contribution of red cell transfusion, increased permeability-oedema and atelectasis to ventilatory and radiographic abnormalities after trauma and (non-cardiovascular) surgery is incompletely understood [6,9,13,16,20-22,24].

To evaluate the relative contribution of the factors in postoperative pulmonary dysfunction, we measured the PLI and EVLW together with haemodynamics, oxygenation, lung mechanics and radiographic abnormalities in mechanically ventilated ALI/ARDS patients admitted to the intensive care unit (ICU) after thoracic trauma and major, non-cardiovascular surgery.

## Methods

This prospective study was approved by the Ethical Committee of the Vrije Universiteit medical centre and written informed consent was obtained from each patient or relatives. We included various consecutive trauma/surgery groups since comparing the groups would allow to judge the relative role of red cell transfusion and increased permeability-oedema versus atelectasis in pulmonary dysfunction and ALI/ARDS of various surgical aetiologies. Consecutive, mechanically ventilated patients within 24 h after direct, blunt thoracic trauma needing chest tube drainage were included. Consecutive patients after major surgery were included within 12 h after transhiatal oesophagectomy (via an upper abdominal incision), abdominal cancer surgery (via median laparotomy) or major bone surgery, following preoperative written informed consent. Inclusion criteria were the presence of radial artery and central venous catheters and the absence of overhydration, defined by a CVP >11 or >14 mmHg if positive end-expiratory pressure (PEEP) >10 cm H_2_O, since the latter may elevate atmospheric pressure-referenced intrathoracic filling pressures, measured at end-expiration, by PEEP. Exclusion criteria were an age of 78 years or above, pregnancy and a life expectancy less than 24 h. Radial artery and central venous catheters were used for haemodynamic measurements and blood sampling. The lungs were volume-controlled ventilated with a tidal volume (V_t_) of 6–8 ml kg^-1 ^resulting in an end-tidal CO_2_-concentration between 4 and 5%, using an O_2_-air mixture and PEEP of 5 cm H_2_O or more, when needed (I:E 1:2). We did not routinely attempt to recruit potential atelectatic areas prior to the study.

### Protocol

Because of limited availability of ^67^Ga, the study was performed within 12 hours after completion of surgery or within 24 h after time of trauma. Demographics were recorded, including variables for calculation of the acute physiology and chronic health evaluation (APACHE-II) score and red cell transfusion history (from 12 h prior to study, also for trauma), measurements of PLI, EVLW and haemodynamics were performed, and an anteroposterior chest radiograph was made. Otherwise, only leukocyte-depleted red blood cell concentrates are used in the Netherlands. Haemodynamic variables were measured after calibration and zeroing to atmospheric pressure at mid-chest level (Tramscope^R^, Marquette, Wisc., USA). CVP was taken at end-expiration, with patients in the supine position. Arterial blood samples were obtained for determinations of partial O_2_/CO_2 _pressures and O_2_-saturations (Rapidlab 865, Bayer Diagnostics, Tarrytown, NY, USA, at 37°C). Central venous blood was taken simultaneously for measurement of partial pressures and saturations. Venous admixture was calculated according to standard formulae. The F_I_O_2_, tidal volume, plateau inspiratory pressure and PEEP (cm H_2_O) were taken from the ventilator. Doses of vasoactive drugs were recorded. Patients were taken care of by intensive care physicians not involved in the study and followed until extubation and discharge/death in the ICU.

### Measurements

**Figure 1:**
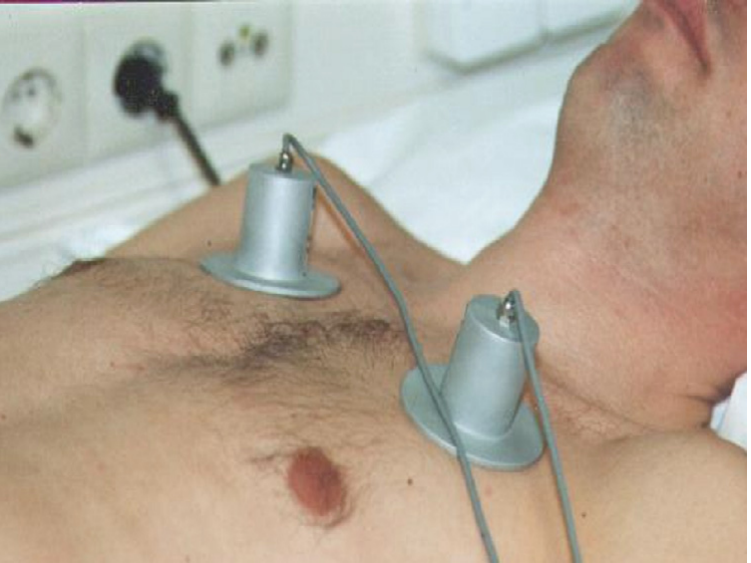
A healthy volunteer with two cesium iodide scilliation probes positioned over the lung apices, for collecting ^67^Ga and ^99m^Tc radioactivity data for calculating the pulmonary leak index (PLI).

The PLI was measured as described before [5]. In brief, autologous red blood cells were labelled with ^99m^Tc (11 MBq, physical half-life 6 h; Mallinckrodt Diagnostica, Petten, The Netherlands). Transferrin was labelled in vivo, following i.v. injection of ^67^Ga-citrate, 4.5 MBq (physical half-life 78 h; Mallinckrodt Diagnostica, Petten, The Netherlands). Patients were in the supine position and two scintillation detection probes (Eurorad C.T.T., Strassburg, France) were positioned over the lungs [Fig. 1; ref [5]]. Starting at the time of injection of ^67^Ga, radioactivity was detected every minute, during 30 minutes. The count rates were expressed as counts per minute (CPM) per lung field. Until 30 minutes after ^67^Ga injection, blood samples (2 ml aliquots) were taken. Each blood sample was weighed and radioactivity was determined by a single well well-counter, corrected for background, spill over and decay (LKB Wallac 1480 WIZARD, Perkin Elmer, Life Science, Zaventem, Belgium). Results were expressed as CPM.g^-1^. For each blood sample, a time-matched CPM over each lung was taken. A radioactivity ratio was calculated, (^67^Ga-lung/^99m^Tc-lung)/(^67^Ga-blood/^99m^Tc-blood), and plotted against time. The PLI was calculated using linear regression analysis and is a measure of pulmonary vascular permeability [5]. The values for both lung fields were averaged, while the upper limit of normal is 14.1 × 10^-3^.min^-1 ^and the measurement error is about 10% [5]. The PLI typically exceeds 40 × 10^-3^.min^-1 ^in ARDS [5].

The EVLW was measured with help of the transpulmonary thermal-dye technique [2-4,7,8,10]. A 4F introducing sheath (Arrow, Reading, USA) was inserted into the femoral artery, for the purpose of the study, in each patient. A 3F fiberoptic thermodilution catheter was inserted in the femoral artery sheath. Fifteen ml of ice cold indocyanine green (ICG), 1 mg.ml^-1 ^dextrose 5%, was injected in a central vein and the thermal-dye dilution curve obtained at the femoral artery (COLD Z-021, Pulsion Medical Systems, Munich, Germany). This allowed calculation of the transpulmonary cardiac output, global end-diastolic volume and the extravascular thermal volume in the lungs as a measure of EVLW (normal <7 ml.kg^-1^) [7]. EVLW is typically two to three-fold elevated in case of overt (radiographic) pulmonary oedema [4]. Measurements were done in duplicate and averaged. The cardiac output and global end-diastolic volume was indexed to body surface area (cardiac index, CI; global end-diastolic volume index, GEDVI) and EVLW to body weight.

The lung injury score (LIS) was calculated from the number of quadrants on the chest radiograph with densities, the PEEP level, the arterial PO_2 _(P_a_O_2_)/inspiratory O_2 _fraction (F_I_O_2_) and the total respiratory dynamic compliance [11,12]. The compliance was calculated from tidal volume/(plateau pressure-PEEP), ml.cm^-1 ^H_2_O. The chest radiograph was scored by a consultant radiologist, blinded to the study, who evaluated the number of quadrants with alveolar densities, ranging from 0 to 4. Signs of basal atelectasis, such as blurring of diaphragm and costophrenic angles and poor visibility of the left-sided diaphragm in the retrocardiac space, were also sought for. The LIS ranges between 0 (no injury) to 4, with values above 2.5 indicative of ARDS, and between 0 and 2.5 of ALI [11,12].

### Statistical analysis

We compared groups with help of the non-parametric Kruskal-Wallis test for continuous and with help of the X^2 ^test for categorical data. We compared the thoracic trauma group with the surgical groups with use of the Mann-Whitney U and Fisher exact tests, while the other surgical groups were compared with help of the Kruskal-Wallis and X^2 ^tests. The Spearman correlation coefficient r_s _was used to express relations. Exact P values > 0.001 are given, while a P < 0.05 was considered statistically significant. All tests are two-sided. Data were summarized as median and range.

## Results

### Patients

Table 1 shows patient characteristics and, as expected, limited comparability of groups of different types of trauma/surgery. All trauma patients had plain radiographic signs of pulmonary contusion and chest tube drainage for haematopneumothorax. Two of them had ARDS (LIS ≥ 2.5), all other patients had ALI (LIS>0). Abdominal surgery included a Whipple procedure for pancreatic carcinoma, partial liver resection for metastases (n = 3), and surgery for rectal and gastric carcinoma. Patients after major bone surgery group had undergone stabilization of the spine (n = 3) or extensive skull surgery (n = 1).

**Table 1:** Patient characteristics

	**Thoracic trauma**	**Oesophageal surgery**	**Abdominal surgery**	**Bone surgery**	**P**
	**n = 5**	**n = 8**	**n = 6**	**n = 4**	

Age (yr)	36 (22–57)^1^	66 (30–77)	66 (55–73)	45 (25–54)^a^	0.006
Sex (men/women)	3 (60)/2 (40)	7 (87)/1 (12)	5 (83)/1(17)	2 (50)/2 (50)	0.435
Weight, kg	85 (7–95)	80 (57–101)	77 (50–97)	77 (58–83)	0.637
Height, m	1.7 (1.6–1.8)	1.8 (1.7–1.9)	1.7 (1.6–1.8)	1.8 (1.6–1.9)	0.588
APACHE II	7 (4–18)	12 (8–16)	10 (7–13)	7.5 (5–11)^a^	0.062
Injury severity score	22 (22–35)	-	-	-	
Chest tube/pleurotomy	5 (100)	6 (75)	0	3 (75)	0.004
Duration of surgery (min)	212 (120–305) (n = 2)	282 (180–630)	205 (80–255)	555 (375–1193)^b^	0.015
Duration mechanical ventilation (h)					
	263 (35–694)^2^	10 (6–70)	17 (10–43)	81 (18–352)	0.010
Length of stay (d)	23 (4–31)^3^	1 (1–3)	1 (1–4)	1 (1–21)	0.007
Time to study (h)	13 (2–24)^4^	2 (1–3)	1.5 (1–2)	2.7 (1–4)	0.024
ICU mortality	0	1 (12)	0	0	0.686

Median (range) or number (percentage), where appropriate. APACHE, acute physiology and chronic health evaluation; ICU, intensive care unit. Versus surgical groups: ^1^P < 0.009; ^2^P = 0.005; ^3^P < 0.001; ^4^P = 0.002; among surgery groups: ^a^P = 0.029; ^b^P = 0.007.

### Haemodynamic and respiratory variables

Table 2 shows the haemodynamic and Table 3 the pulmonary data. The higher CVP in the thoracic trauma group can be explained by higher PEEP, and a higher heart rate may have contributed to a higher CI. An elevated EVLW occurred in a minority (30%) of patients and only 35% of the patients with a LIS ≥ 1 had an EVLW above normal, while groups did not differ. In contrast, the PLI was elevated in 60% of patients after thoracic trauma, in 37% after transhiatal oesophagectomy, in 83% after abdominal surgery and in 100% after bone surgery (P = 0.050). The two trauma patients with ARDS had a PLI of 29 and 13 × 10^-3^.min^-1 ^and an EVLW of 2.3 and 7.6 ml.kg^-1^, respectively. Patients after thoracic trauma showed a higher LIS with elevated pulmonary venous admixture, as compared to the other groups, having only mild pulmonary dysfunction and ALI. Airway pressures were also highest in the thoracic trauma group, at similar tidal volumes. Groups did not differ in the occurrence of radiographic signs of atelectasis. Patients with atelectasis had higher venous admixture (by median 6%, P = 0.036), lower P_a_O_2 _and P_a_O_2_/F_I_O_2 _(by median 126 for the ratio, P = 0.005) and more radiographic densities (P = 0.005) than those without, while the PLI and EVLW did not differ.

**Table 2:** Hemodynamic variables

	**Thoracic trauma**	**Oesophageal surgery**	**Abdominal surgery**	**Bone surgery**	**P**
	**n = 5**	**n = 8**	**n = 6**	**n = 4**	

Heart rate (bpm)	97 (89–108)^1^	56 (44–82)	66 (46–89)	60 (49–104)	0.014
MAP (mmHg)	78 (67–84)	87 (60–113)	81 (75–100)	74 (65–105)	0.763
CVP (mmHg)	9 (7–14)^2^	4 (0–6)	3 (1–5)	4.5 (2–7)	0.007
GEDVI (ml.m^-2^)	617 (492–1125)	823 (574–1068)	726 (521–922)	751 (380–826)	0.668
CI (l.min^-1^.m^-2^)	5.8 (3.8–8.2)^3^	2.8 (2.2–3.9)	3.2 (2.4–3.5)	2.9 (2.0–5.5)	0.056
Dopamine (mg.h^-1^)	0 (0–40)	8 (0–32)	0 (0–8)	1.5 (0–4)	0.442
Red cell concentrates	0 (0–3)	0 (0–12)	3.5 (2–7)	9 (0–32)	0.133

Median (range) or number of patients, where appropriate. MAP, mean arterial pressure; CVP, central venous pressure; GEDVI (n 680–800 ml^-1^.m^2^), global end-diastolic volume index; CI, cardiac index. Versus surgical groups ^1^P = 0.001; ^2^P < 0.001; ^3^P = 0.003; ^4^P = 0.005; among surgical groups ^a^P = 0.008.

**Table 3:** Respiratory variables

	**Thoracic trauma**	**Oesphageal surgery**	**Abdominal surgery**	**Bone surgery**	**P**
	**n = 5**	**n = 8**	**n = 6**	**n = 4**	

PLI (×10^-3^.min^-1^)	26 (12–29)	12 (6–38)	49 (20–106)	71 (16–171)	0.073
PLI >14.1 (x10^-3^.min^-1^)	3 (60)	3 (37)	5 (83)	4 (100)^a^	0.050
EVLW (ml.kg^-1^)	3.4 (2.3–7.6)	5.6 (2.9–11.6)	6.0 (3.5–10.3)	6.0 (2.9–8.7)	0.433
EVLW >7 (ml.kg^-1^)	1 (20)	2 (25)	2 (33)	2 (50)	0.861
P_a_O_2 _(mmHg)	121 (57–147)^1^	140 (97–211)	176 (156–234)	192 (176–229)^a^	0.004
P_a_CO_2 _(mmHg)	45 (22–56)	35 (27–41)	33 (26–36)	37 (31–38)	0.209
F_I_O_2 _(%)	70 (40–88)	41 (40–59)	40 (39–49)	42 (40–48)	0.230
P_a_O_2_/F_I_O_2_	173 (78–359)^2^	328 (188–515)	439 (390–510)	458 (414–515)^b^	0.003
S_a_O_2 _(%)	98 (92–98)	98 (97–99)	98 (98–99)	98 (98–99)	0.186
Q_s_/Q_t _(%)	27 (21–57)^3^	15 (6–40)	13 (8–18)	16 (7–21)	0.022
RR (/min)	19 (15–26)^4^	14 (11–16)	14 (11–16)	15 (11–19)	0.045
Tidal volume (ml.kg^-1^)	6.7 (4.4–8.8)	7.3 (6.2–9.3)	7.3 (6.3–8.9)	7.9 (7.5–9.8)	0.327
Plateau pressure (cm H_2_O)					
	28 (20–34)^3^	16 (12–22)	15 (13–19)	16 (15–17)	0.015
PEEP (cm H_2_O)	12 (11–16)^3^	6 (4–8)	5 (4–6)	7.5 (6–9)	0.002
Compliance_*tot*.,*resp*.,*dyn*.,_					
ml.cm^-1 ^H_2_O	31 (19–93)	59 (46–97)	62 (32–76)	66 (57–108)	0.296
Quadrants with densities				
	1 (0–4)	1 (0–2)	0 (0–2)	1 (0–1)	0.456
Radiographic atelectasis				
	3 (60)	5 (62)	1 (17)	1 (25)	0.257
Lung injury score	2.2 (1.5–3.5)^3^	0.7 (0.5–1.7)	0.5 (0.2–0.7)	0.7 (0.5–1.0)	0.015

PLI, pulmonary leak index; EVLW, extravascular lung water; PO_2_/PCO_2_, partial pressure of O_2 _or CO_2_; F_I_O_2_, inspiratory O_2 _fraction; SO_2_, O_2 _saturation (a = arterial); Q_s_/Q_t_, venous admixture; RR, respiratory rate; PEEP, positive end-expiratory pressure; tot. resp. dyn. total respiratory dynamic. Exact P if <0.05. Versus surgical groups: ^1^P = 0.007, ^2^P = 0.004; ^3^P ≤ 0.001; ^4^P = 0.004; among surgical groups ^a^P = 0.019; ^b^P = 0.020.

### Correlations

**Figure 2:**
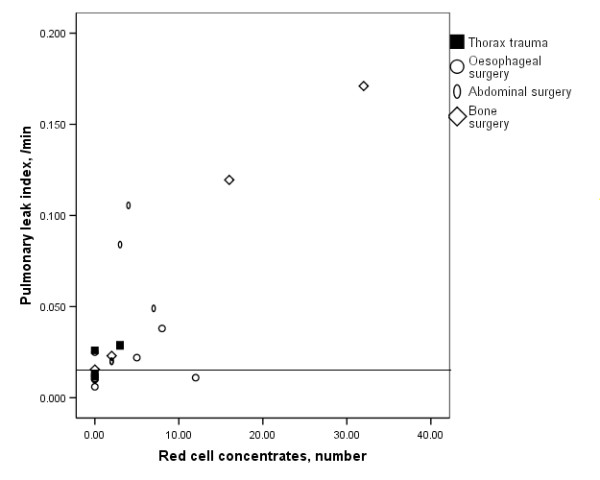
Relation between number of red cell concentrates administered and the pulmonary leak index (PLI, × 10^-3^.min^-1^) in the patient groups: r_s _= 0.69, P < 0.001. The upper limit of normal PLI is 14.1 ×10^-3^.min^-1^.

**Figure 3:**
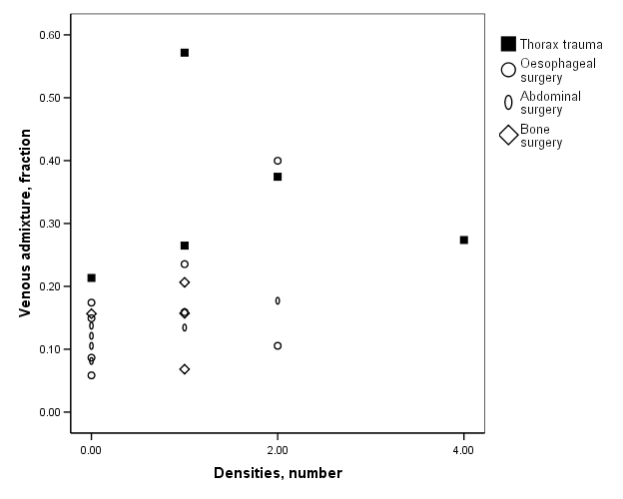
Relation between number of plain radiographic quadrants with densities versus venous admixture: r_s _= 0.55, P = 0.007. Similar relations were present with P_a_O_2 _and P _a_O_2_/F_I_O_2 _(r_s _= -0.52, P = 0.011).

The PLI directly related to the number of red cell concentrates given prior to the study (r_s _= 0.69, P = < 0.001, Fig. 2). The venous admixture and oxygenation related to the number of quadrants with densities on plain radiography (Fig. 3). Neither the PLI nor the EVLW related to any ventilatory or radiographic variable.

### Course

The length of stay in the ICU directly related to the PEEP level (r_s _= 0.61, P = 0.002) and was thus longer in the direct trauma than in surgical groups. One oesophagectomy patient died in the ICU.

## Discussion

Our data, obtained in surgical, mechanically ventilated patients with pulmonary dysfunction, indicate that impaired oxygenation is associated with radiographic densities, relatively independent of transfusion-related increased permeability-oedema and type of trauma/surgery.

We used the LIS to define ALI/ARDS and grade pulmonary dysfunction, in the absence overhydration in our patients, since the definition takes the effect of PEEP into account, in contrast to the consensus conference definition. Indeed, ventilatory abnormalities may better predict duration of mechanical ventilatory support after trauma than consensus conference ALI/ARDS criteria [12,17,18,30]. Nevertheless, both definitions of ALI/ARDS and their severity may be insensitive and non-specific for increased permeability-oedema; ALI/ARDS may not be accompanied by an elevated EVLW in about one third of patients, and the contribution of atelectasis to increased permeability-oedema in ALI/ARDS is increasingly recognized [1,6,9,14,15,18,30,31]. We therefore sought to study the relative contribution of increased permeability-oedema and signs of atelectasis to oxygenation impairment and radiographic densities in surgical ALI/ARDS patients. We used the non-invasive PLI, since ALI/ARDS is typically accompanied by a 2 to 6-fold increase in PLI and the latter is of high value for discrimination from cardiogenic oedema [12], and the EVLW, since this is the only bedside method for direct assessment of pulmonary oedema [2-4,7,8,10].

We observed a high PLI in the majority of abdominal and, particularly, bone surgery patients, which related to extensive red cell transfusion (Fig. 2), in agreement with the literature suggesting an elevated TRALI risk in extensive bone and spinal surgery [25,32,34]. We cannot exclude, however, that the relation was not direct, as in TRALI [27,28], and reflected a common underlying factor, such as shock, systemic ischemia/reperfusion and severity of trauma [28]. An elevated PLI in the absence of severe pulmonary oedema and high EVLW after surgery, which agrees with other studies [2,3], suggests that adjustment of Starling forces, alveolar resorption and/or increased pulmonary lymph flow had offset alveolar oedema formation by increased permeability [1,30]. Moreover, the PLI increase may have been transient, as after major cardiovascular surgery [2,3].

Pulmonary dysfunction and ALI/ARDS after thoracic trauma was associated with a greater oxygenation defect and higher LIS than in patients with extrapulmonary injury, irrespective of increased permeability-oedema. Hence, we can attribute increased venous admixture, at least in part, to atelectasis, even though direct lung injury has been suggested to be associated with less atelectasis and alveolar recruitability than indirect injury [6,9,14,16]. Conversely, the plain radiographic densities of lung contusions may have been caused, at least in part, by atelectasis. Other authors also described a relation between impaired oxygenation and radiographic abnormalities after lung contusion [17], but this is controversial [18]. That lung contusion is only partly associated with lung injury and (permeability) oedema is supported by a poor relation between plain radiographic densities and EVLW reported before [4], and an increase in aeration (CT scanning) of dependent areas upon high airway pressures or the prone position, suggesting opening of atelectatic alveoli [15]. Indeed, pulmonary contusion may alter surfactant and thereby predispose to atelectasis [19]. The worse oxygenation and higher airway pressures needed to maintain oxygenation, at similar PLI, EVLW and tidal volume, after thoracic trauma than major surgery may thus indicate greater ventilation to perfusion mismatching in atelectatic areas associated with densities on the plain radiograph. Mismatching may have been facilitated by a higher CI promoting venous admixture. Otherwise, the ventilatory abnormalities following the direct thoracic injury partly determined the duration of mechanical ventilatory support in our study, as in others [12,17,18,30], rather than increased permeability-oedema.

Patients undergoing transhiatal oesophageal, abdominal and bone surgery served as contrast groups for thoracic trauma, in spite of limited comparability, since surgery may carry postoperative pulmonary complications involving indirect or remote pulmonary injury [13,16,20-26,34]. Indeed, our patients had mild ALI with somewhat impaired oxygenation and total respiratory dynamic compliance and plain radiographic densities, irrespective of PLI and EVLW, and thus attributable again, at least in part, to atelectasis [9]. This may agree with the literature, showing a normal PLI in many patients after transthoracic oesophagectomy, in spite of increased circulating proinflammatory factors and occurrence of ALI/ARDS [21]. The mild ALI observed after abdominal surgery may also be attributable, at least in part, to atelectasis. Indeed, basal atelectasis and resultant pulmonary radiographic densities is a major mechanism of hypoxaemia after anaesthesia and abdominal surgery even though increased permeability oedema may contribute to postoperative ALI [9,13,16,23,24,29]. In spite of having the highest PLI, patients after bone and spinal surgery did not exhibit more pulmonary oedema nor greater impairment of gas exchange as compared to other surgical groups, again suggesting that oxygenation impairment and pulmonary densities were not caused by increased permeability-oedema alone.

Limitations of our study include the relatively low numbers of each surgical group, limited comparability and a difference in timing, so that conclusions should be drawn cautiously. In fact, the manifestations of pulmonary trauma may take hours to develop. The measurement of the PLI may carry a sampling error and the measurement of EVLW may depend on regional perfusion and thermal accessibility may be limited in direct lung injury [8,10]. Hence, we cannot exclude that we have underestimated the occurrence and extent of increased permeability-oedema in the thoracic trauma group. The central venous blood used for calculation of venous admixture in this study may not substitute for mixed venous blood, if mixing in the right atrium is incomplete [33]. Nevertheless, changes in O_2 _saturation, rather than absolute values, in central venous blood may well correlate to those in mixed venous blood [33]. Even though CT scanning may better visualize pulmonary contusions [18] and dorsal atelectasis, developing during anaesthesia and surgery, than plain radiographs [9], we did not perform CT scanning since this involves transportation. Moreover, interpretation may be hard and does not always allow unequivocal differentiation of oedema from atelectasis. We did not routinely apply recruitment manoeuvers to verify if pulmonary densities are indeed caused by recruitable atelectasis, in the absence of a standard reference method [9,13-16].

## Conclusion

The oxygenation defect and radiographic densities of pulmonary dysfunction and ALI/ARDS after thoracic trauma and major surgery are not primarily caused by permeability-oedema relating to red cell transfusion. This suggests a contribution by atelectasis, even after thoracic trauma.

## Pre-publication history

The pre-publication history for this paper can be accessed here:


